# Coxsackievirus B3 Activates Macrophages Independently of CAR-Mediated Viral Entry

**DOI:** 10.3390/v16091456

**Published:** 2024-09-13

**Authors:** Yasir Mohamud, Jingfei Carly Lin, Sinwoo Wendy Hwang, Amirhossein Bahreyni, Zhihan Claire Wang, Honglin Luo

**Affiliations:** 1 Centre for Heart Lung Innovation, University of British Columbia, St. Paul’s Hospital, Vancouver, BC V6Z 1Y6, Canada; 2 Department of Pathology and Laboratory Medicine, University of British Columbia, Vancouver, BC V6Z 1Y6, Canada

**Keywords:** macrophages, coxsackievirus B3 (CVB3), coxsackievirus and adenovirus receptor (CAR), LC3, innate immunity

## Abstract

Enteroviruses are a genus of small RNA viruses that are responsible for approximately one billion global infections annually. These infections range in severity from the common cold and flu-like symptoms to more severe diseases, such as viral myocarditis, pancreatitis, and neurological disorders, that continue to pose a global health challenge with limited therapeutic strategies currently available. In the current study, we sought to understand the interaction between coxsackievirus B3 (CVB3), which is a model enterovirus, and macrophage cells, as there is limited understanding of how this virus interacts with macrophage innate immune cells. Our study demonstrated that CVB3 can robustly activate macrophages without apparent viral replication in these cells. We also showed that myeloid cells lacked the viral entry receptor coxsackievirus and adenovirus receptor (CAR). However, the expression of exogenous CAR in RAW264.7 macrophages was unable to overcome the viral replication deficit. Interestingly, the CAR expression was associated with altered inflammatory responses during prolonged infection. Additionally, we identified the autophagy protein LC3 as a novel stimulus for macrophage activation. These findings provide new insights into the mechanisms of CVB3-induced macrophage activation and its implications for viral pathogenesis.

## 1. Introduction

Enteroviruses, which are a genus of small RNA viruses, are responsible for approximately one billion global infections annually. These infections range in severity from the common cold and flu-like symptoms to more severe diseases, such as viral myocarditis, pancreatitis, and neurological disorders [1]. With the exception of poliovirus, which has nearly been eradicated in much of the globe, non-polio enteroviruses continue to pose a global health challenge, with limited therapeutic strategies currently available.

Coxsackievirus B3 (CVB3), which is a cardiotropic enterovirus, is a small, positive-sense RNA virus with a single-stranded RNA genome that encodes 11 viral proteins: 4 structural capsid proteins and 7 non-structural proteins [2,3,4]. Among these, the RNA-dependent RNA polymerase (viral protein 3D) and two viral proteinases (2A and 3C) play crucial roles in viral RNA production and viral polyprotein processing, respectively. Additionally, these pathogenic factors can interface with other host factors to contribute to viral pathogenesis [5,6].

Viruses in general exhibit unique tropisms for various cell types due to factors such as the expression of specific viral entry receptors, such as the expression of the coxsackievirus and adenovirus receptor (CAR), and the presence or absence of cell-specific factors that influence viral replication [7]. Innate immune cells, such as macrophages, respond to infections through pathogen recognition receptors (PRRs). Among these, toll-like receptors (TLRs) are a key family of PRRs that detect viral components [8]. For instance, TLR3 recognizes double-stranded RNA, while TLR7 and TLR8 detect single-stranded RNA, which triggers antiviral responses [8]. These receptors activate signaling pathways, including the NF-κB pathway, which plays a pivotal role in the inflammatory response to infections [9]. However, viral infection also elicits host cellular injury and the release of damage-associated molecular patterns (DAMPs) that can also initiate inflammatory signals.

In the current study, we sought to understand the interaction between CVB3 and macrophage cells, as there is limited understanding of how this virus interacts with macrophage innate immune cells. While extensive research explored the interactions of cardiotropic viruses, like CVB3, with cardiomyocytes, fewer studies investigated the potential pathological involvement of macrophages. Our study demonstrated that CVB3 infection can robustly activate macrophages without apparent viral replication in these cells. We showed that myeloid cells lacked the viral entry receptor CAR and were defective in viral replication, even after the introduction of exogenous CAR. We also identified the autophagy protein microtubule-associated protein 1 light chain 3 (MAP1LC3/LC3) as a novel DAMP that can stimulate macrophage activation. This finding provides new insights into the mechanisms of CVB3-induced macrophage activation and its implications for viral pathogenesis.

## 2. Materials and Methods

### 2.1. Cell Culture and Viral Infection

An HL-1 cell line (mouse atrial cardiomyocyte) was a kind gift from W. Claycomb (Louisiana State University Medical Center, New Orleans, LA, USA) and cultured in Claycomb medium (Millipore Sigma, St. Louis, MO, USA, cat# 51800C) supplemented with 4 mM glutamine, 100 μM norepinephrine, and 10% fetal bovine serum (FBS). THP-1 human monocyte cell line, was a kind gift from G. Francis (Centre for Heart Lung Innovation, University of British Columbia) and cultured in RPMI-1640 medium supplemented with 10% FBS. For differentiation into macrophages, THP-1 suspension cells were incubated overnight with 500 nM of phorbol 12-myristate-13-acetate (PMA). RAW 264.7 mouse macrophage cells, HeLa and HEK293 cells (American Type Culture Collection, Manassas, VA, USA, TIB-71CCL-2 and CRL-1573) were cultured in Dulbecco’s Modified Eagle’s Medium (DMEM) supplemented with 10% FBS and a penicillin–streptomycin cocktail (100 µg/mL). SIM-A9 mouse microglia cells (ATCC, Cat#CRL-3265) were a kind gift from Dr. Neil Cashman (University of British Columbia) and cultured in DMEM:F12 medium with 10% FBS. NSC-34 motoneuron-like cells were a kind gift from Dr. Neil Cashman (University of British Columbia) and cultured in DMEM supplemented with 10% FBS. For the CVB3 infection, the cells were either sham infected with PBS (Sigma-Oakville, Oakville, ON, Canada, D8537) or inoculated with CVB3 (Kandolf strain; generously provided by Reinhard Kandolf, University of Tubingen, Tubingen, Germany) at different MOIs, as specified in the figure legends.

### 2.2. Chemical Treatment

The following chemicals were used for the treatment of the cells: STING agonist, 2′3′-cGAMP (Invivogen, San Diego, CA, USA, tlrl-nacga23-02), or diABZI (Cayman Chemical, Ann Arbor, MI, USA, 28054) for 4 h at the indicated concentrations outlined in the respective figure legends. The cGAS and STING inhibitors, RU.521 (Cayman Chemical, cat#31765), and H-151 (Invivogen, cat# inh-h151) were used at 10 μM in the control or CVB3-infected RAW264.7 cells. The following TLR inhibitors were used in this study: TLR1/2, CU-CPT22 (Cayman Chemicals, cat# 15244), TLR3-inhibitor, CU-CPT4A (Cayman Chemicals, cat#30951), TLR4-inhibitor C34 (Cayman Chemicals, cat#18515), or NFkB inhibitor peptide (Cayman Chemicals, cat#17493) at the concentrations indicated in the respective figure legends. Recombinant human LC3 (rLC3) was purchased from R&D Systems (cat# UL-430) and used at the concentrations indicated in the figures.

### 2.3. Immunofluorescence and Confocal Microscopy

RAW 264.7 or HeLa cells were cultured in 8-well chamber slides (Thermofisher Scientific, Burnaby, BC, Canada, 155411) for 24 h prior to the treatments. After the treatments, the cells were fixed at 37 °C for 30 min in a 4% paraformaldehyde/100 mM sucrose fixative. Formaldehyde was quenched with three rinses of 0.1 M glycine/PBS, and the cells were permeabilized with 0.1% Triton X-100 (Sigma, T8787) for 3 min, followed by 1 h of blocking in 3% BSA (Sigma, A7030)/PBS. The cells were incubated overnight with primary antibody diluted in 3% BSA/PBS following the manufacturer’s dilution recommendation. Upon rinsing the primary antibody with PBS (3 washes of 5 min each), the cells were incubated for an additional 1 h in Alexa Fluor conjugated secondary antibody followed by subsequent rinses as above. The cells were mounted with Fluoroshield and 4,6-diamidino-2-phenylindole (DAPI; Sigma-Aldrich, St. Louis, MO, USA, F6057). Immunofluorescence images were captured on an EVOS M5000 and confocal images were captured with a Zeiss LSM 880 Inverted Confocal Microscope using a 63× objective lens.

### 2.4. Western Blot Analysis

Western blot analysis was performed as previously described [10]. Briefly, the cells were lysed on ice with MOSLB buffer (10 mM HEPES, pH 7.4, 50 mM NaPyrophosphate, 50 mM NaF, 50 mM NaCl, 5 mM EDTA, 5 mM EGTA, 100 µM Na_3_VO_4_, 0.1% Triton X-100). The lysates were spun at 13,000 rpm to pellet the debris, and approximately 30 μg of protein was loaded and subjected to SDS-PAGE. Western blotting was conducted using the following primary antibodies: Cox2 (D5H5) (Cell Signaling Technology, Danvers, MA, USA, #13314), p-TBK1 (Cell Signaling Technology, #5483), TBK1 (Cell Signaling Technology, #3504), STING (Cell Signaling Technology, #13647), p-STING (Cell Signaling Technology, #19781), VP1 (Mediagnost, Cox mAb 31A2), ACTB (Sigma-Aldrich, A5316), LC3B (Novus Biologicals, Centennial, CO, USA, NB100-2220), and CAR (Cell Signaling Technology, #16984).

### 2.5. Real-Time Quantitative RT-PCR (RT-qPCR)

Total RNA was extracted using the Monarch^®^ Total RNA Miniprep Kit (New England Biolabs, Whitby, ON, Canada, #T2010S). To determine the gene expression levels, qPCR targeting of the genes *IFNB*, *IL6*, *CXCL10*, *CCL5*, *IL-1B* and *TNFA* was performed in a 10 µL reaction containing 1 μg of RNA using the Luna^®^ Universal One-Step RT-qPCR kit (New England Biolabs, #E3005L) and normalized to *ACTB* mRNA according to the manufacturer’s instructions. The PCR reaction was performed on a ViiA 7 Real-Time PCR System (Applied Biosystems, Waltham, MA, USA). The samples were run in biological triplicates and analyzed using the comparative CT (2^−ΔΔCT^) method with control samples and the results are presented as relative quantitation (RQ). The primer sequences for the RT-qPCR are provided in the table below (Table 1).

### 2.6. Lentivirus Production

Lentiviruses that expressed GFP control or human CAR (hCAR) were created as previously described [11]. Briefly, GFP and hCAR sequences were commercially generated through the services of Integrated DNA technologies^®^ and subcloned into pLenti-MP2 plasmid (AddGene, Watertown, MA, USA, plasmid #36097). The following packaging (psPAX2; plasmid #12260), envelope (pMD2.G; AddGene, plasmid #12259), and Rev plasmids (pRSV-Rev; AddGene, plasmid #12253) were used for the lentiviral production. Constructs were transfected into 293 T Lenti-X (Takara Bio, Kusatsu, Japan, Cat# 632180) for 72 h prior to viral harvesting. The supernatants that contained viruses were concentrated using the Amicon^®^ Ultra Centrifugal Filter, 100 kDa MWCO (Millipore, Burlington, MA, USA, Cat# UFC9100). Concentrated viruses were aliquoted and stored at −80 °C.

### 2.7. Quantification and Statistical Analysis

Statistical analysis was performed using GraphPad Prism 5 or Microsoft Excel software (version v16.61.1). Statistical differences between the two groups were calculated using an unpaired Student’s *t*-test. A comparison of multiple groups was statistically evaluated with analysis of variance (ANOVA) and Tukey’s post hoc test.

## 3. Results

### 3.1. CVB3 Induced Pro-Inflammatory Gene Expression in Macrophages Independent of Viral Replication

**Figure 1:**
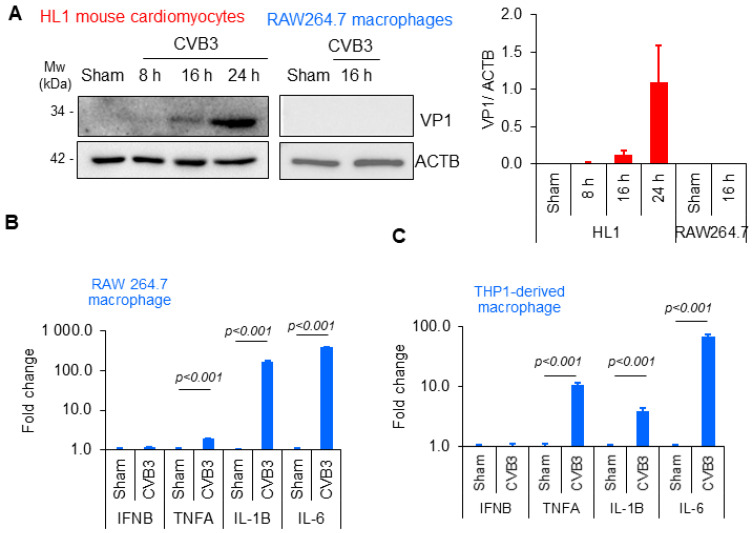
CVB3 induced pro-inflammatory gene expression in macrophages independent of the viral replication. (**A**) HL1 cardiomyocytes or RAW264.7 macrophage cells were subjected to CVB3 infection (MOI = 100) for the indicated timepoints. Lysates were subjected to Western analysis for the viral capsid protein (VP1) and normalized to the loading control ACTB. Densitometric analysis results are shown in the adjacent bar plot. (**B**) RAW264.7 murine macrophages and (**C**) THP1-derived human macrophages were infected with CVB3 (MOI = 10; 4 h). RNA was harvested and subjected to qPCR analysis for inflammatory gene markers IFNB, TNFA, IL-1B, and IL-6. Viral gene marker 2A was utilized as a control to assess the viral treatment. The results are presented as the relative gene fold change expression between sham- and CVB3-infected groups (mean ± S.D., n = 3), where they were statistically evaluated via an unpaired Student’s *t*-test.

To elucidate the mechanism of cell-type specific interactions with CVB3, we utilized the murine cardiomyocyte cell line HL1 and murine macrophage cell line RAW264.7. Both cell lines were infected with CVB3 at a multiplicity of infection (MOI) of 100, which is a viral dose that was previously shown to facilitate robust infection [12]. Cell lysates were harvested at 16 h and 24 h post-infection and subjected to Western blot analysis to assess the viral replication through the detection of the viral capsid protein VP1. Intriguingly, unlike the HL1 cells, which demonstrated viral protein production at 16 h, the RAW264.7 macrophage cells did not produce VP1 following infection (Figure 1A).

We next sought to determine whether these innate immune cells could mount a response to viral infection. To this end, the RAW264.7 cells or macrophages derived from human THP1 monocytes were cultured with CVB3 (MOI = 10; 4 h). RNA was purified and subjected to quantitative RT-PCR to measure key inflammatory genes, including interferon β (IFN β), tumor necrosis factor alpha (TNF-α), interleukin 1 beta (IL-1β), and interleukin 6 (IL-6), which were normalized to the housekeeping gene ACTB. Of note, despite their inability to produce viral proteins, both macrophage cell types tested demonstrated robust pro-inflammatory gene expression (Figure 1B,C). Collectively, this evidence demonstrates that CVB3 is capable of inducing robust pro-inflammatory signaling in macrophages without the production of viral proteins.

### 3.2. CVB3-Induced Macrophage Activation Was Independent of cGAS-STING Pathway

**Figure 2:**
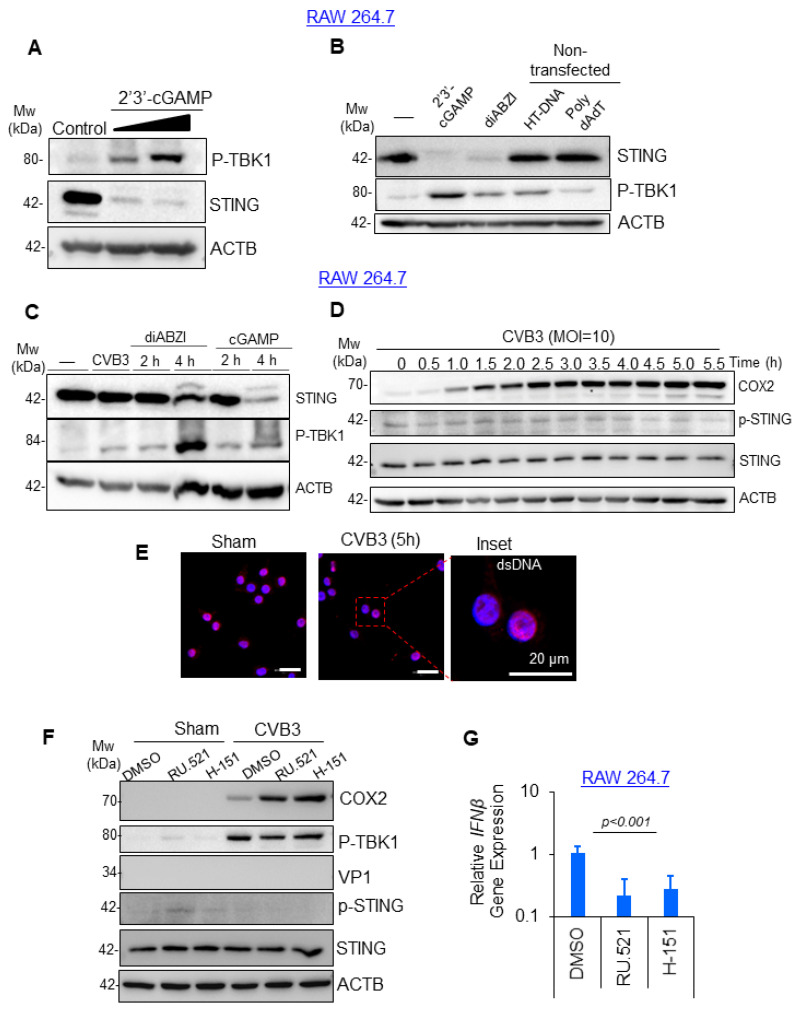
CVB3-induced macrophage activation was independent of the cGAS-STING pathway. (**A**) RAW264.7 murine macrophages were subjected to a dose ramp of the STING agonist 2′3′-cGAMP (10 and 20 μg/mL, 4 h) and subjected to Western analysis for STING and p-TBK1 protein expression; ACTB was used as the loading control. (**B**) RAW264.7 macrophages were treated with STING agonists 2′3′-cGAMP (20 ug/mL) and diABZI (10 μM) or cGAS agonists herring testis (HT)-DNA (3 μg/mL) or poly-dAdT (3 μg/mL). cGAS-STING pathway activation was assessed via Western analysis of the STING and p-TBK1 protein expressions. (**C**) STING was not activated following CVB3 infection. RAW264.7 cells were infected with CVB3 (MOI = 10; 4 h) as above, and lysates were harvested for Western analysis of the STING activation, p-TBK1, and ACTB loading control. The RAW264.7 cells were subjected to a timecourse STING agonist treatment with diABZI (10 μM), and cGAMP (32 μM) was the positive control. (**D**) RAW264.7 macrophages were subjected to a time-course infection with CVB3 (MOI = 10), and cell lysates were analyzed by Western blot for macrophage activation marker COX2 and cGAS-STING pathway activation (anti-STING, anti-p-STING). ACTB was used as the loading control. (**E**) RAW264.7 macrophages were infected with CVB3 (MOI = 10; 5 h) and the cells were fixed for confocal microscopy imaging of the dsDNA/mtDNA release. Scale bars: 20 µm. (**F**) RAW264.7 macrophages were sham or CVB3 infected (MOI = 10; 8 h) in the presence of cGAS and STING inhibitors RU.521 (10 μM) and H151 (10 μM), respectively. Cell lysates were subjected to Western analysis for macrophage activation (COX2), STING activation (anti-STING, anti-p-STING, anti p-TBK1), and viral replication (VP1). (**G**) RAW264.7 macrophages were treated with cGAS and STING inhibitors RU.521 (10 μM) and H-151 (10 μM) as above, and the drug efficacy was evaluated with a qPCR assessment of the relative IFNB gene expression (mean ± S.D., n = 3).

Given that the RAW264.7 macrophages responded robustly to viral treatment in the absence of viral protein production, we next sought to identify the mechanism of macrophage activation. Previous studies linked RNA viruses and the DNA-sensing cGAS-STING innate immune pathway [13,14]. Therefore, we rationalized that viral injury established in viral-permissive cells, such as cardiomyocytes, may release DAMPS that directly activate cGAS-STING. cGAS acts as a cytosolic sensor of mislocalized or pathogen-associated DNA and responds by synthesizing the secondary messenger molecule 2′3′cGAMP, which can bind and activate the downstream adaptor protein STING. To evaluate the role of cGAS-STING in CVB3-mediated macrophage activation, we treated RAW264.7 cells with 2′3′cGAMP and assessed the downstream signaling molecules STING and TBK1. Indeed, the RAW264.7 cells treated with 2′3′cGAMP demonstrated robust activation of STING and TBK1 phosphorylation in a dose-dependent manner, thus providing evidence that the pathway was intact in this cell line (Figure 2A). Next, we compared various cGAS and STING agonists with their relative strengths in pathway activation in the RAW264.7 cells. Interestingly, 2′3′cGAMP and the potent non-nucleotide STING agonist diamidobenzimidazoles (diABZI) robustly activated STING and TBK1 phosphorylation (Figure 2B). However, the treatment of RAW264.7 cells with the cGAS agonists herring testis (HT) DNA or poly-dAdT did not significantly activate STING despite modestly activating downstream TBK1. Using the established chemicals as a positive control, we tested whether CVB3 infection could stimulate STING activation. RAW264.7 cells were infected with CVB3 (MOI = 10; 4 h) alongside control groups stimulated with the STING agonists diABZI or cGAMP after 2 or 4 h of treatment. Intriguingly, CVB3 infection was not associated with the STING activation, whereas treatment with the positive controls diABZI and cGAMP robustly activated STING following 4 h of treatment (Figure 2C). To test the potential for transient STING activation that could be missed during single timepoint infections, the RAW264.7 macrophages were subjected to a time course CVB3 infection experiment. The RAW264.7 cell lysates were collected in 30 min intervals following CVB3 infection and analyzed by Western blotting for cyclooxygenase 2 (COX2) expression as a marker of macrophage activation, as well as the STING and p-STING expressions to assess cGAS-STING activation. Indeed, the CVB3 infection robustly activated macrophages as early as 1 h post-infection with sustained COX2 expression detected at late infections (Figure 2D). Surprisingly, the CVB3 infection was not associated with any detectable activation of STING at all timepoints tested. Consistent with this, the CVB3-infected RAW264.7 macrophages did not demonstrate significant alterations in dsDNA staining suggestive of mislocalized DNA when compared with the sham-infected controls (Figure 2E). Moreover, the treatment of RAW264.7 cells with the cGAS or STING chemical inhibitors RU.521 and H-151, respectively, following the CVB3 infection had no effect on the COX2 expression or downstream TBK1 activation (Figure 2F). The efficacy of the cGAS-STING pathway inhibition by RU.521 and H-151 was tested in the RAW264.7 cells, which demonstrated robust inhibitory effects of downstream inflammatory cytokine IFNβ, as assessed by qPCR (Figure 2G). Consistent with our earlier observation, we were unable to detect any viral protein production (VP1) in the RAW264.7 cells at all timepoints or dosages of CVB3 infection tested in this study. Altogether, this evidence supports the idea that the CVB3-mediated activation of RAW264.7 macrophages is likely independent of the cGAS-STING pathway.

### 3.3. CVB3-Induced Macrophage Activation Is Dependent on NF-κB

**Figure 3:**
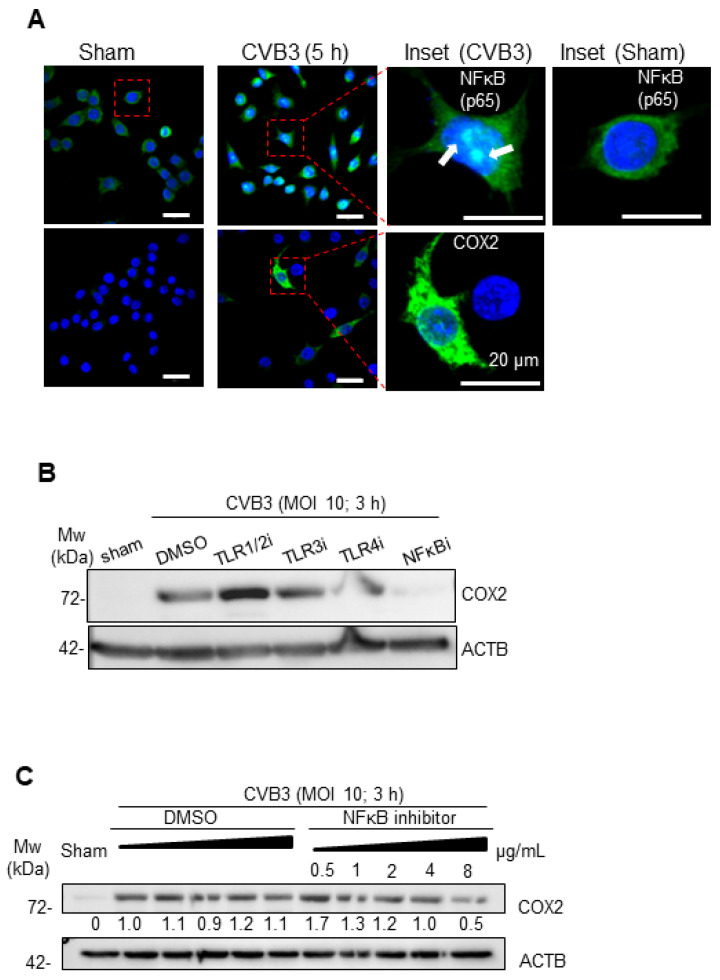
CVB3-induced macrophage activation was dependent on NF-κB (**A**) RAW 264.7 macrophages were infected with CVB3 (MOI = 10; 5 h), and the cells were fixed for confocal microscopy imaging of NFkB activation and COX2 expression. Scale bars: 20 µm. White arrows denote the nuclear-localized p65 present in CVB3- but not sham-infected cells. (**B**) RAW264.7 macrophages were infected with CVB3 (MOI = 10; 3 h) and subjected to specific inhibitors of TLR1/2, CU-CPT22 (500 nM), TLR3-inhibitor, CU-CPT4A (27 μM), TLR4-inhibitor C34 (10 μM), or NFkB inhibitor peptide (50 ug/mL). Cell lysates were subjected to Western analysis for the macrophage activation marker (COX2). (**C**) RAW264.7 macrophages were infected as in (**A**) and subjected to a dose ramp of the NFkB peptide inhibitor. Lysates were subjected to Western analysis for the macrophage activation marker COX2.

After excluding the cGAS-STING pathway in the CVB3-mediated macrophage activation, we explored the potential involvement of other inflammatory pathways. Among the markers of macrophage activation tested in our study, we reliably observed COX2 protein expression following CVB3 treatment. Given that COX2 is a downstream target of the nuclear factor kappa B (NFκB) pathway, we examined the localization of this transcription factor in the CVB3-infected RAW264.7 cells. Indeed, the CVB3-infected RAW264.7 cells demonstrated a strong nuclear localization of NFκB, as well as downstream COX2 protein expression (Figure 3A).

To examine which upstream pathway may be activated by CVB3 to initiate NFκB signaling, we assessed several extracellular TLRs previously linked to viral protein/RNA sensing, including TLRs 1/2, 3, and 4 [8]. Interestingly, a selective blockade of TLR1/2, TLR3, or TLR4 with chemical inhibitors did not significantly alter the CVB3-induced COX2 production in the RAW264.7 cells, whereas the positive control, namely, a peptide inhibitor of NFkB, robustly abrogated the COX2 induction in a dose-dependent manner (Figure 3B,C). Taken together, these studies illustrate that CVB3-mediated macrophage activation was dependent on the NFκB pathway.

### 3.4. CVB3-Induced Macrophage Activation Is Independent of Viral Replication, CAR Expression, and Phagocytosis

**Figure 4:**
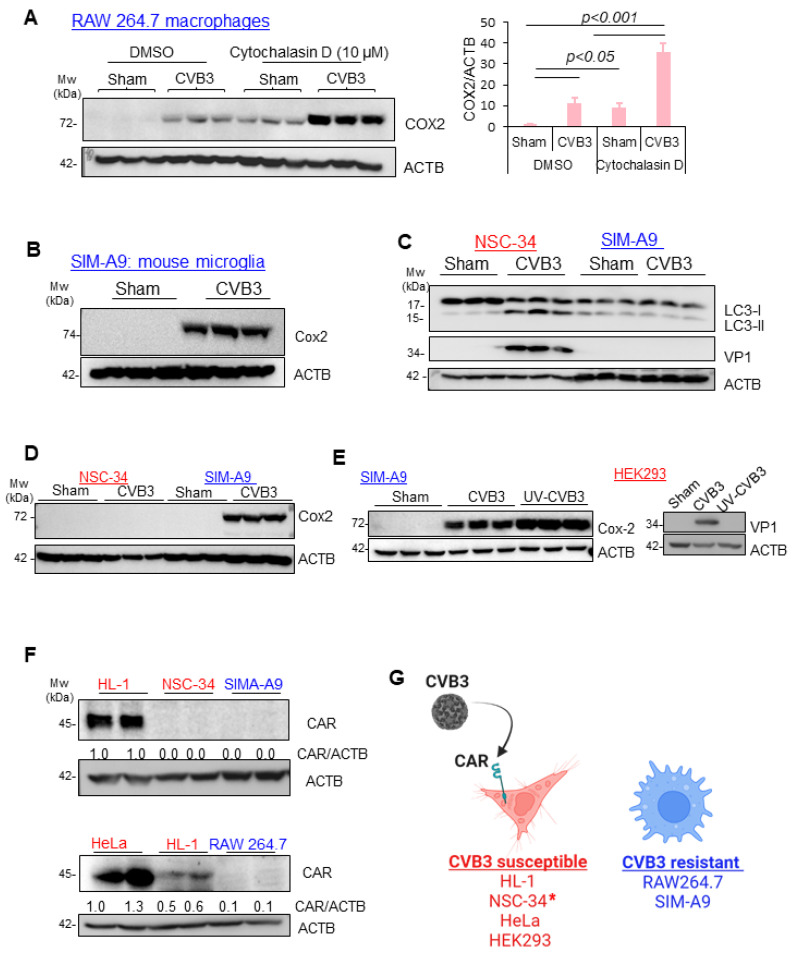
CVB3-induced macrophage activation was independent of the viral replication, CAR expression, and phagocytosis. (**A**) RAW264.7 macrophages were sham or CVB3 infected (MOI = 10; 8 h) in the presence of the phagocytosis inhibitor cytochalasin D (1 μM) or a DMSO control. Cell lysates were assessed by Western blotting for the macrophage activation marker COX2. Densitometry results of the COX2 activation are presented in the right-hand panel (mean ± S.D., n = 3). (**B**) The SIM-A9 murine microglia cell line was assessed for myeloid activation through Western analysis with a COX2 marker following sham or CVB3 infection as above. (**C**) CVB3 replication and virus-induced autophagy was assessed in the NSC-34 murine motor neuron cells and SIM-A9 macrophages (MOI = 10; 8 h). Lysates were subjected to Western analysis for the autophagy marker protein LC3 and viral replication marker VP1. (**D**) The specificity of the COX2 myeloid expression marker was assessed in the NSC-34 motor neuron cells and SIM-A9 microglia cell line through Western analysis in the presence or absence of CVB3 infection (MOI = 10; 8 h). ACTB served as the loading control. (**E**) Virus-induced COX2 expression in SIM-A9 microglia was assessed with a control CVB3 virus and UV-inactivated CVB3 (MOI = 10; 8 h). UV inactivation of CVB3 was verified in the CVB3-susceptible cell line HEK293 through Western analysis for the viral replication marker VP1. (**F**) Expression of the coxsackievirus entry receptor CAR was assessed in HL-1, NSC-34, SIM-A9, HeLa, and RAW264.7 cells through Western analysis with the anti-CAR antibody. ACTB served as a protein loading control. (**G**) Schematic illustration of CVB3-susceptible cells and CVB3-resistant cells. * Denotes NSC-34 as the only CVB3-susceptible cell line that did not have detectable CAR expression.

To elucidate the mechanism of CVB3-induced macrophage activation, we sought to better study their direct interaction. As phagocytic cells, macrophages can ingest various pathogens, including viruses, for immune clearance. We first inquired whether CVB3-mediated macrophage activation is dependent on its phagocytic function. Cytochalasin D is a fungi-derived mycotoxin that inhibits phagocytosis by disrupting the polymerization of actin filaments, which are essential for the formation of the phagocytic cup [15]. The RAW264.7 macrophages were infected with CVB3 or sham infected in the presence or absence of cytochalasin D. The cell lysates were subjected to Western blot analysis to assess the COX2 expression. Intriguingly, the COX2 expression following the CVB3 infection was further enhanced in the presence of cytochalasin D treatment (Figure 4A). To assess the generalizability of our observations toward myeloid cell types, we also investigated the murine microglia cell line SIM-A9. Similar to the RAW264.7 cells, SIM-A9 microglia infected with CVB3 robustly activated the COX2 expression (Figure 4B) despite the absence of viral protein production (Figure 4C). As a positive control, NSC-34 motor-neuron-like cells were also tested for CVB3 susceptibility and demonstrated a strong induction of VP1 and virus-replication-induced autophagy (Figure 4C), whereas the COX2 expression was exclusive to the myeloid cell types examined in this study (Figure 4D). Given that viral protein was undetectable in the SIM-A9, we examined whether COX2 induction was dependent on the replication capacity of the virus. We compared the replication-competent CVB3 with a UV-irradiated preparation. Interestingly, both the control and UV-inactivated CVB3 could robustly induce COX2 expression in the SIM-A9. The CVB3-susceptible cell line HEK293 was used to verify the UV-inactivation efficacy, as no detectable viral protein was observed with the UV-CVB3 infection (Figure 4E). Lastly, we assessed whether the cell types investigated in this study expressed the viral entry receptor, namely, the coxsackievirus and adenovirus receptor (CAR). Of note, among the cell types examined, only the HL1 cardiomyocytes and HEK293/HeLa cells, which served as the positive controls, expressed CAR, whereas the RAW264.7 macrophages and SIM-A9 microglia cell types did not express CAR. Interestingly, NSC-34 was the only susceptible cell line that lacked any detectable expression of the viral entry receptor CAR (Figure 4F,G). Collectively, this evidence suggests that susceptibility to CVB3 infection was largely dependent on the expression of the viral entry receptor CAR, which was absent in the myeloid cells RAW264.7 and SIM-A9 evaluated in this study.

### 3.5. CAR Expression Regulates Late, but Not Early, Response to CVB3 Infection

**Figure 5:**
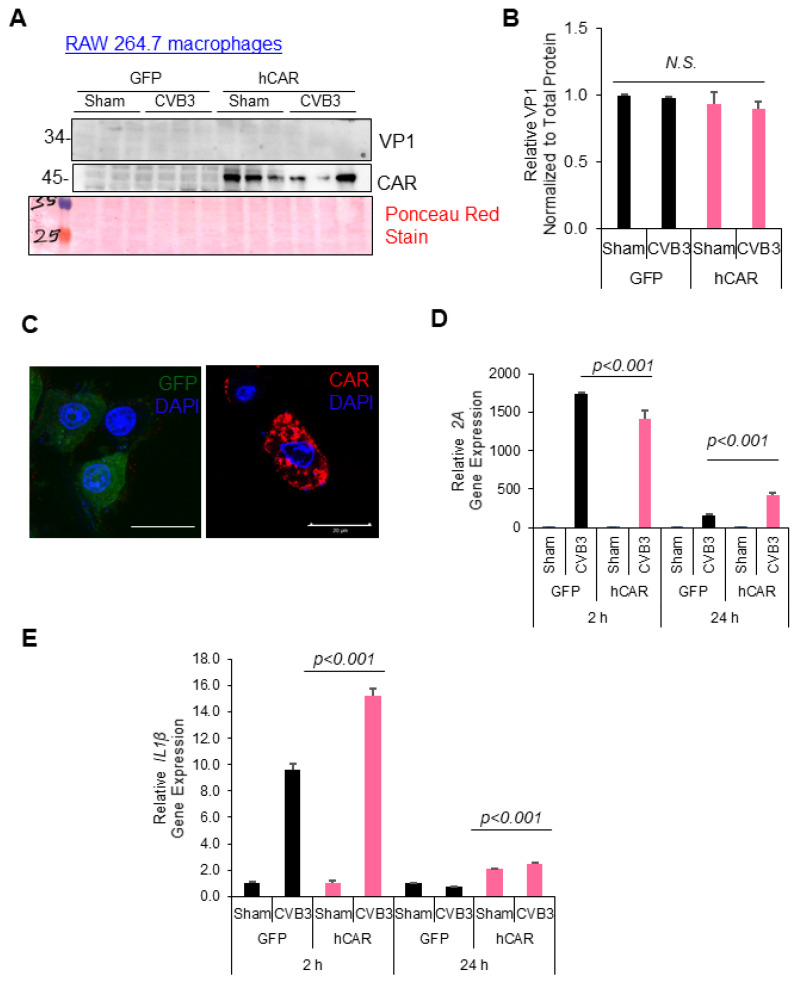
CAR expression regulated the late, but not early, response to the CVB3 infection. (**A**) RAW264.7 cells were transduced with either a control GFP- or hCAR-expressing lentivirus for 72 h, followed by CVB3 infection (MOI = 10) for 24 h. Cell lysates were harvested and subjected to Western analysis for anti-VP1 and anti-CAR antibody. Ponceau Red was used as a total protein loading stain. (**B**) Densitometry of VP1 expression from (**A**) was quantified and normalized to the total protein and presented in the bar plot (mean ± S.D., n = 3). (**C**) RAW264.7 cells were transduced as above and subjected to confocal analysis for GFP (green) and CAR (red) expression. Nuclei were stained with DAPI (blue). Scale bar: 20 µm. (**D**,**E**) RAW264.7 cells transduced with a GFP or hCAR lentivirus as above were subsequently infected with CVB3 (MOI = 10) for 2 h (**D**) or 24 h (**E**). RNA was harvested and subjected to qPCR analysis for viral RNA (2A) replication or inflammatory markers IL-1β, IL-6, or TNFA and presented as a relative quantitation where the first group was set to 1 (mean ± S.D., n = 3).

Given that the myeloid cell types investigated in this study did not express CAR, we sought to evaluate whether the introduction of human CAR (hCAR) could render them susceptible to productive viral infections. To this end, the RAW264.7 cells were transduced with either a GFP control or hCAR expressing a lentivirus for 72 h, followed by CVB3 infection (MOI = 10) for 24 h. As expected, the transduction of RAW264.7 cells with a lentivirus that encoded for hCAR resulted in robust expression following 72 h of infection. Interestingly, the hCAR-expressing RAW264.7 cells did not show productive viral infection, as assessed by Western analysis for VP1 (Figure 5A,B). To assess the functionality of the hCAR expressed in the RAW264.7 cells, we performed confocal microscopy to image the hCAR localization in the transduced RAW264.7 cells. Indeed, the hCAR was robustly expressed in the RAW264.7 cells that were predominantly localized to the cell periphery (Figure 5C). To further assess the functionality of the hCAR in the RAW264.7 cells, we examined viral RNA at two timepoints following CVB3 treatment consistent with early (2 h) and late (24 h) infection periods. Interestingly, there was a minor reduction in the viral uptake in the hCAR-expressing cells at the early timepoint; however, viral RNA was significantly elevated in the hCAR-transduced RAW264.7 cells at late infection (Figure 5D,E). Additionally, no significant differences in the inflammatory gene (IL-1β, TNFA) expression were observed between the hCAR- and control GFP-expressing cells at the early timepoint of infection (Figure 5D). Of note, the late infection was associated with significantly increased inflammatory gene expression and viral RNA in the hCAR-transduced cells. Together, this evidence suggests that the CAR expression in the RAW264.7 cells could modulate viral and host inflammatory gene expressions without significantly impacting the viral propagation.

### 3.6. Viral-Induced LC3 Stimulates Macrophage Activation

**Figure 6:**
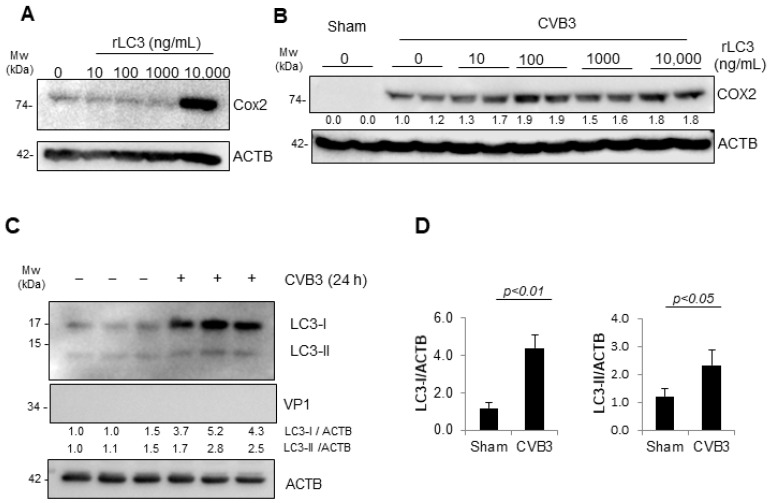
Viral-induced LC3 stimulated macrophage activation. (**A**) Recombinant LC3 (rLC3) activated the SIMA-A9 myeloid cells. SIM-A9 were incubated with increasing doses of rLC3 for 4 h. Lysates were harvested and subjected to Western analysis for COX2 expression. ACTB was used as a loading control. (**B**) rLC3 potentiated CVB3-induced activation of the SIM-A9. SIM-A9 cells were co-incubated with CVB3 (MOI = 10) and increasing doses of rLC3 for 4 h. Lysates were harvested as above and subjected to Western analysis (**C**) SIM-A9 cells were infected with CVB3 (MOI = 10; 24 h). Lysates were harvested and subjected to Western analysis for autophagy marker LC3 and normalized to ACTB. (**D**) Densitometry of panel (**C**) where LC3-I and LC3-II were quantified and normalized to ACTB and presented as the mean ± S.D., n = 3.

Given that the early kinetics of macrophage activation following the CVB3 infection was largely CAR-independent, we rationalized that viral replication may not be a major determinant of early macrophage activation. Beyond the traditional PAMPs, such as viral nucleic acids and proteins, host-derived DAMPs have been recognized as significant factors in the activation of innate immune pathways [16]. We therefore rationalized that DAMPs present alongside CVB3 during transmission may be possible stimulus candidates for myeloid cells. A growing body of research has provided supporting evidence that EVs hijack the host autophagy pathway for viral propagation and dissemination [3,17,18]. In particular, CVB3 was shown to escape cells in extracellular vesicles and/or mitochondria-associated vesicles that stain positive for the autophagy marker protein microtubule-associated protein 1 light chain 3 (MAP1LC3/LC3). To investigate the possible involvement of LC3 in the innate immune activation of myeloid cells, we first treated the murine myeloid cell line SIM-A9 in media supplemented with recombinant purified LC3 protein at increasing dosages for 4 h. The cell lysates were harvested and subjected to Western analysis for the macrophage activation marker COX2. Of interest, we observed significant COX2 expression in the SIM-A9 cells treated with LC3 (10 ug/mL) (Figure 6A). We next asked whether rLC3 could potentiate the macrophage stimulatory properties of CVB3. To this end, the SIM-A9 cells were infected with CVB3 in the presence of increasing concentrations of rLC3 for 4 h. Interestingly, the co-treatment with CVB3 and rLC3 significantly increased the COX2 induction (Figure 6B). Finally, we assessed the downstream autophagy pathway of the SIM-A9 cells following the CVB3 infection. As we previously did not observe significant alternations in the LC3 protein expression following the 8 h infection of SIM-A9 cells, we performed a longer 24 h infection. Indeed, this prolonged infection of SIM-A9 cells was associated with increased LC3-I and LC3-II protein expression in the absence of viral protein production (Figure 6C,D). Together this evidence suggests that LC3 can act as a DAMP to activate SIM-A9.

## 4. Discussion

Macrophages are innate immune sentinel cells that guard and protect against pathogen invasion by recognizing and responding to infections. With respect to coxsackievirus infection, macrophages were shown to play important roles in disease pathogenesis [19,20,21,22]. For example, Li et al. demonstrated that differential macrophage polarization in male vs. female mice is linked to a viral myocarditis disease outcome [23].

Our study identified that despite cardiac cells being susceptible to CVB3 and myeloid cells being resistant, it was macrophages that primarily mediated the virus-induced inflammatory responses. A likely contributing factor was the differential expression of the viral entry receptor CAR between these cell types. CAR is an epithelial tight junction and cardiac intercalated disc protein [24]. Indeed, differential expressions of CAR across tissues and organ systems has been noted as a strategy for CVB3-based therapeutic strategies [25,26]. Although CVB3 was shown to replicate in distinct immune cell populations, including B and T lymphocytes, it is unclear whether such replication occurs in macrophages [27,28]. Interestingly, Shin et al. recently tested the role of CAR in macrophages and found a potential involvement of CAR in the regulation of innate immunity [20]. Although in our studies, CAR expression was undetectable in the RAW264.7 at the protein level, similar to the findings of Lindner et al. [29], Shin and colleagues demonstrated that the treatment of RAW264.7 macrophages with LPS or TNFα could stimulate CAR expression [20]. Consistent with the findings of Lindner et al., our study showed that CVB3 did not have a productive infection in RAW264.7 macrophages. Furthermore, our study demonstrated that the introduction of CAR into macrophages could not rescue the viral replication deficits. Given their specialized roles in innate immunity and their robust immune reactivity to viral infection, macrophages may be uniquely equipped with defense mechanisms, including an abundance of receptors for sensing damage and pathogen-associated molecular patterns to overcome viral entry [30,31].

Using two different myeloid cell types, our study also demonstrated that the CVB3 treatment robustly induced macrophage activation independent of viral replication. We utilized UV-irradiated CVB3 that was replication defective to exclude the requirement for viral replication. Consistent with our findings, Bao et al. showed that RAW264.7 activation is independent of CVB3 replication [22]. Interestingly, the authors observed a modest upregulation of NLRP3 expression upon the transfection of RAW264.7 cells with viral capsid proteins VP1/2. Given that NLRP3 is downstream of NFκB, it is plausible that CVB3 viral capsids may have been involved in the activation of the NFκB pathway in our study. The ability of CVB3 to induce pro-inflammatory responses independent of viral replication parallels observations in other viral infections, where immune responses are triggered by viral components rather than active replication [29,32].

Beyond PAMPs, host-derived DAMPs can act as powerful stimulants to innate immune cells, but the precise host factors that initiate pathological inflammation remains an actively investigated area [16]. In the current study, we tested whether host-derived DAMPs, such as mislocalized DNA, may be a mechanism of CVB3-induced RAW264.7 activation. Interestingly, CVB3 infection was not associated with activation of the cytosolic DNA sensor cGAS-STING pathway in the RAW264.7 cells. Consistent with this, the cGAS-STING pathway was recently shown to not be activated in CVB3-replicative cells [14]. With respect to CVB3 non-replicative cells, such as RAW264.7 macrophages, the absence of cGAS-STING activation likely suggests a lack of mislocalized cytosolic DNA. Indeed, the CVB3 infection of RAW264.7 cells in our study was not associated with cytosolic dsDNA staining. We previously showed that EVs, such as CVB3 and EV-D68, block an important cellular recycling and quality control process called autophagy [18,33]. Autophagy is a housekeeping process that is essential for the clearance of both PAMPs and DAMPs during infections by many pathogens [34,35]. Interestingly, EVs were shown to hijack the host autophagy pathway to facilitate efficient viral replication and propagation [3,36,37,38]. In our current study, we demonstrated that the autophagy protein LC3, which is typically anchored to autophagic vesicles, can act as a stimulant to macrophages. Indeed, previous studies provided evidence for the release of LC3 and LC3-associated vesicles from CVB3-infected cells [39,40,41]. We posit that in addition to viral PAMPs, macrophages may also elicit inflammatory responses to host-derived DAMPs, such as LC3, which is typically restricted to intracellular compartments, but may be secreted upon infection. Moreover, we observed the induction of LC3-I and LC3-II following prolonged infection of the myeloid cell line. One possibility is that prolonged infection may have activated a non-canonical autophagy process called LC3-associated phagocytosis (LAP), which facilitates the enhanced killing and degradation of ingested pathogens [42]. Consistent with this, we observed a strong downregulation of viral RNA in both the control and hCAR-transduced macrophages following prolonged infection, which suggests a mechanism of clearance may become active in the late stages of infection.

Collectively, our study underscored the complex interplay between viral infections and innate immune responses that contribute to the broader narrative of how viruses exploit host cellular machinery. These insights may open avenues for targeted therapies that can modulate specific inflammatory pathways, thus offering potential benefits in managing viral-related inflammatory diseases.

## Figures and Tables

**Table 1:** Primer sequences for RT-qPCR used in this study.

	Forward	Reverse
2A	GCTTTGCAGACATCCGTGATC	CAAGCTGTGTTCCACATAGTCCTTCA
Ifnb1 (mouse)	GCCTTTGCCATCCAAGAGATGC	ACACTGTCTGCTGGTGGAGTTC
IFNB (human)	CAACTTGCTTGGATTCCTACAAAG	TATTCAAGCCTCCCATTCAATTG
Il6 (mouse)	ACAACCACGGCCTTCCCTAC	TCTCATTTCCACGATTTCCCAG
IL6 (human)	ACTCACCTCTTCAGAACGAATTG	CCATCTTTGGAAGGTTCAGGTTG
Il1b (mouse)	GCAACTGTTCCTGAACTCAACT	ATCTTTTGGGGTCCGTCAACT
IL-1B (human)	CCACAGACCTTCCAGGAGAATG	GTGCAGTTCAGTGATCGTACAGG
Tnfa (mouse)	GTCCCCAAAGGGATGAGAAGTT	GTTTGCTACGACGTGGGCTACA
TNFA (human)	CCTCTCTCTAATCAGCCCTCTG	GAGGACCTGGGAGTAGATGAG

## Data Availability

Data are available for sharing upon request to the corresponding author.
